# Event-Related Potentials Reveal Rapid Verification of Predicted Visual Input

**DOI:** 10.1371/journal.pone.0005047

**Published:** 2009-03-31

**Authors:** Michael Dambacher, Martin Rolfs, Kristin Göllner, Reinhold Kliegl, Arthur M. Jacobs

**Affiliations:** 1 Department of Psychology, Universität Potsdam, Potsdam, Germany; 2 Department of Education and Psychology, Freie Universität Berlin, Berlin, Germany; 3 Laboratoire Psychologie de la Perception, CNRS - Université Paris Descartes, Paris, France; Macquarie University, Australia

## Abstract

Human information processing depends critically on continuous predictions about upcoming events, but the temporal convergence of expectancy-based top-down and input-driven bottom-up streams is poorly understood. We show that, during reading, event-related potentials differ between exposure to highly predictable and unpredictable words no later than 90 ms after visual input. This result suggests an extremely rapid comparison of expected and incoming visual information and gives an upper temporal bound for theories of top-down and bottom-up interactions in object recognition.

## Introduction

Perception is not the result of passive bottom-up transmission of physical input [1]. Instead active top-down projections continuously interact with earliest stages of sensory analysis. This insight increasingly influences our understanding of cognitive efficiency [2]–[5]. For instance, attention enhances neural responses to visual stimuli in extrastriate and striate visual cortices [6], and already on the subcortical level in the LGN [7]. In fact, studies using functional magnetic resonance imaging (fMRI) revealed modulations in cortical and subcortical areas even *prior* to sensory input of expected stimuli [7]–[9]. We regard such anticipatory activity as top-down predictions engaging lower-level areas involved in sensory processing to grant fast and smooth perception of forthcoming stimuli. Given that the quantity of feedback connections to primary sensory areas even outnumbers pure feedforward input [5] the interplay of top-down and bottom-up flow appears as a major principle of perception.

Beyond fMRI-based evidence about spatial characteristics of neural activity, temporal information contributes to the comprehension of bottom-up and top-down processes. Employing the high temporal resolution of electroencephalography (EEG), research predominantly focused the influence of attention on the time course of visual perception [10]. For instance, spatial attention modulates alpha band activity over occipital areas prior to the appearance of an expected target [11], [12]. After stimulus onset amplitudes on the P1 component evolving at around 70 ms are enhanced for stimuli appearing at attended compared to unattended locations [13]–[16]. Influences of object- and feature-based attention have typically been observed later with a post-stimulus onset at 100 to 150 ms [17]–[22].

However, despite the undisputed role for top-down control, attention cannot be equated with feedback flow per se. Gilbert and Sigman [4] expanded the traditional concept of *attention*-based top-down influences and denominated *expectations* and *perceptual task* as further forms. Although these concepts are strongly overlapping and can hardly be separated, the critical distinction lies in the amount of information top-down streams carry. For example, directing attention to a certain location presumably contains less information than a task affording predictions about the identity of an upcoming stimulus at that position. In particular, strong expectations of a certain stimulus may involve a form of hypothesis testing that compares characteristics of the incoming signal to stored representations even prior to object identification [4]. This idea is implemented in models integrating bottom-up and top-down processes, such that feedforward streams transmitting sensory information converge with feedback activity carrying knowledge and hypotheses about stimuli. For instance, McClelland and Rumelhart [23], [24] proposed that word identification is driven by the interaction of linguistic and context-based knowledge with incoming featural information. Indeed, the amount of top-down feedback can be quantified at the level of individual participants [25]. Grossberg [26] suggested that stimulus-related signals are enhanced, when top-down predictions are correct and match sensory inputs (cf., [27]–[30]). According to such theories, the congruence of prediction and input facilitates stimulus processing, potentially at early perceptual levels. An open question is, however, at what point in time perception benefits from the comparison of top-down and bottom-up processes, when strong predictions are involved.

The present study used event-related potentials (ERPs) to delineate the earliest interaction between expectations about the identity of incoming signals and input-driven information in visual word recognition. Sentence reading is perfectly suited to investigate the issue. As a well-overlearned everyday activity, it involves highly optimized object recognition processes ranging from individual letters and sublexical units to whole words, thereby engaging both early and higher levels of the visual system [31]. Critically, earliest visual cortices were found to be selectively sensitive to trained, letter-like shapes [32]. Furthermore, during normal reading, rapid input rates of four to five words per second require high perceptual efficiency and encourage fast stimulus processing. This is crucial since modulations of early sensory processes are primarily engaged, when task demands and perceptual load are high [10], [33], [34]. Finally, sentence contexts afford strong and form-specific predictions for upcoming words. Indeed, increased neural activity was measured on articles (i.e., a/an) when their phonological form mismatched the initial phoneme of a highly predictable but not yet presented noun (e.g., airplane/kite [35]; see also [36]).

We manipulated predictability of target words in sentences to investigate at what point in time after visual onset expectations about upcoming stimuli are verified. To push the necessity of efficient visual processing and to measure neural responses under near-normal conditions, words were presented at a high rate approximating natural reading speed [37], [38]. Provided that match and mismatch of stimulus and prediction evoke distinct neural responses [5], an early difference between ERPs for predictable and unpredictable words represents an upper bound for the latency of top-down and bottom-up interactions.

## Materials and Methods

### Participants

Thirty-two native German readers (24 female; 29 right-handed; mean age: 27.3, SD: 6.8), recruited at Freie Universität Berlin, received course credit for participation. They had normal or corrected-to-normal vision and reported no history of neurological diseases. The experiment was performed in accordance with the ethical standards laid down in the 1964 Declaration of Helsinki. In agreement with the ethics and safety guidelines at the Freie Universität Berlin, we obtained a verbal informed consent statement from all individuals prior to their participation in the study. Potential participants were informed of their right to abstain from participation in the study or to withdraw consent to participate at any time without reprisal.

### Materials

**Figure 1:**
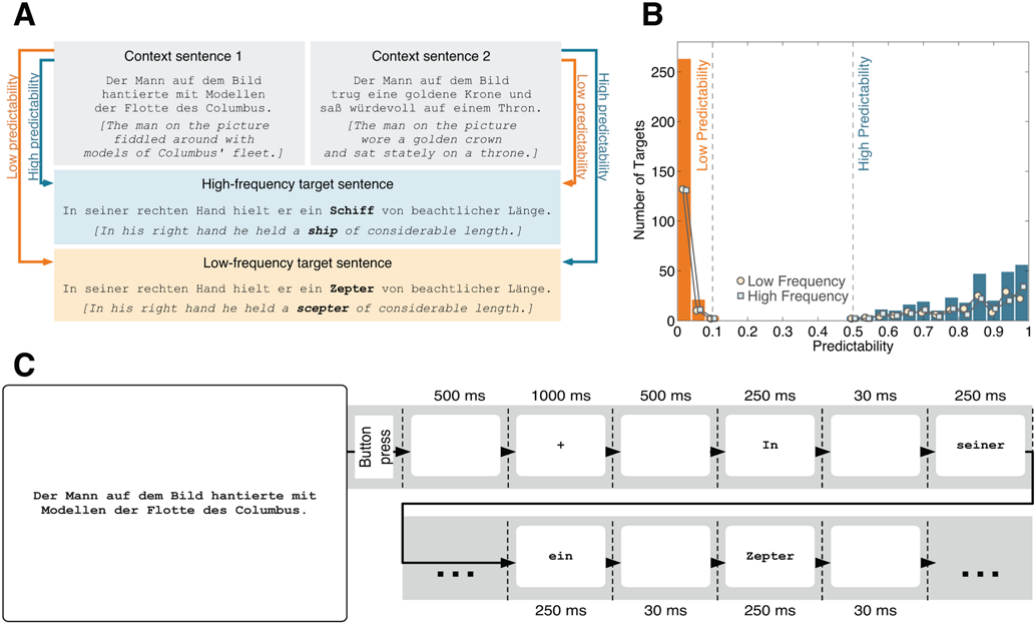
Stimuli and procedure. (A) Stimulus example. High (*ship*) and low frequency (*scepter*) targets were embedded in a neutral sentence frame. Two context sentences triggered low or high predictability of target words. (B) Distribution of predictability values. Bars illustrate the distribution of target predictability across the stimulus material. Low predictability targets (orange) include cloze probabilities no larger than .1. High predictability words (blue) comprise cloze values of at least .5. Lines reflect the dispersion of predictability norms within low (light orange circles) and high frequency (light blue squares) categories. Note that the entire corpus comprises a total of 576 predictability values, since each of the 144 sentence units involves a low and a high frequency target that both serve as low and as high predictability word. (C) Presentation sequence. A context sentence was fully displayed until participants pressed a button. After a fixation cross, the neutral sentence was presented word by word at monitor center. Each word was displayed for 250 ms and followed by a 30 ms blank screen.

A total of 144 sentence units formed the stimulus materials. Each unit comprised two context sentences and one neutral sentence. The latter was identical across conditions except for target words setting up a two-by-two factorial design of frequency and predictability (Figure 1A).

144 pairs of high (e.g., Schiff *[ship]*) and low frequency (e.g., Zepter *[scepter]*) open-class words served as targets. High frequency words comprised lemma and word form frequencies greater than 100 and 10 occurrences per million, respectively. For low frequency words, lemma and word form frequencies were lower than 10 per million. Frequency norms were taken from the DWDS data base [39]. High and low frequency words from one pair were members of the same class (i.e., nouns, verbs, or adjectives) and, where possible, shared the same number of letters; they differed in one letter in 19 of the 144 cases, in two letters in 4 cases and in three letters in 1 case. Target length varied between three and eight letters and was matched across conditions.

Target pairs were embedded at the sixth to eighth word position in neutral sentence frames and were always followed by at least two more words. Two context clauses preceding the neutral sentences triggered predictability of target words: High frequency targets were of high predictability in context 1 and of low predictability in context 2. For low frequency targets the pattern was reversed. Predictability norms were assessed in an independent cloze task performed by a total of 151 voluntary participants; none of them took part in the EEG experiment. In the cloze procedure, a context sentence was presented together with words of the corresponding neutral sentence up to the position prior to the target. Participants then guessed the word that would most likely continue the sentence fragment. They were asked to write at least one, but no more than three guesses per sentence. Each participant was presented with only one context per sentence unit and worked through a part of the stimuli. In total, every sentence was rated by at least 30 subjects. Predictability was computed as the proportion of participants correctly predicting the target word with one of their guesses. In the 144 sentence units entering the stimulus materials both low and high frequency words reached cloze values of at least 0.5 in the high predictability conditions while not exceeding 0.1 in the low predictability conditions. Target word statistics are depicted in Table 1.

**Table 1:** Descriptive statistics of target words.

	LF-LP		LF-HP		HF-LP		HF-HP	
	Mean	SD	Mean	SD	Mean	SD	Mean	SD
**Word form freq.**	3.76	2.08	3.76	2.08	155.58	194.63	155.58	194.63
**Lemma freq.**	4.87	2.68	4.87	2.68	362.19	875.30	362.19	875.30
**Predictability**	.01	.02	.83	.13	.01	.02	.84	.13
**Length**	5.32	1.11	5.32	1.11	5.36	1.16	5.36	1.16
**Word position**	6.94	.76	6.94	.76	6.94	0.76	6.94	.76

Target word norms [mean and standard deviation (SD)] according to the 2×2 experimental manipulation of frequency (low: LF; high: HF) and predictability (low: LP; high: HP). 144 target word pairs consisted of 92 noun-, 37 verb-, and 15 adjective-pairs.

Figure 1B illustrates the distribution of predictability values in the categories. Most low predictability targets had cloze values of zero; in the high predictability condition the number of targets increased with predictability. Cloze values were similarly distributed for low and high frequency words.

For the ERP study, randomized stimuli were divided into lists such that each participant was presented with every sentence unit only once. A Latin square design provided that each version of a sentence unit was presented to the equal number of participants. This resulted in 72 high and 72 low predictability trials per subject, with 36 high and low frequency words in either category.

### Procedure

Participants were seated at a distance of 60 cm from the monitor in a dimly lit room and were asked to silently read two-sentence stories for comprehension. A trial started with a context sentence that was displayed in its entirety until subjects pressed a button. Thereafter, a fixation cross, preceded and followed by a 500 ms blank interval, indicated for 1000 ms the required fixation position at monitor center. The stimuli of the neutral sentence together with their adjacent punctuation were then presented word by word with a stimulus onset asynchrony (SOA) of 280 ms (i.e., stimulus: 250 ms; blank: 30 ms). The presentation sequence of context and neutral sentences is schematized in Figure 1C. After the neutral sentence, either the next trial was initiated (66.67%) or a three-alternative multiple-choice question was inserted to test sentence comprehension (33.33%). Questions referred to the content either of the context or the neutral sentence, but were never related to the target word.

Participants were asked to avoid eye movements and blinks during the interval of word-wise sentence presentation. After eight practice trials and 72 sentence units of the main experiment, they took a short break. Stimuli (font: Courier New; size: 18 pt) were presented in black on a white background.

### Electrophysiological recording and data processing

EEG data were recorded from 50 scalp locations corresponding to the 10/20 international system. Impedances were kept below 10 kΩ. All scalp electrodes and one channel on the right mastoid originally referenced to the left mastoid were re-referenced offline to the average of scalp electrodes. Two horizontal and two vertical EOG electrodes recorded bipolarly oculomotor signals and blinks. Data continuously recorded with a sampling rate of 512 Hz were re-sampled offline to 256 Hz. Amplifier settings cut off frequencies below .01 and above 100 Hz. Data were bandpass filtered offline from .1 to 30 Hz (24 dB; 50 Hz notch).

EEG data contaminated by muscular artifacts and drifts were rejected offline via visual inspection. Independent component analysis (Vision Analyzer, Brain Products GmbH, Germany) was used to remove oculomotor artifacts and blinks. Additionally, an automatic algorithm rejected segments with an absolute amplitude larger than 90 µV in at least one channel. The rejection procedure resulted in the exclusion of 3.17% of all target intervals (low frequency – low predictability: 2.78%; low frequency – high predictability: 2.17%; high frequency – low predictability: 3.82%; high frequency – high predictability: 3.91%). In the remaining data, the continuous EEG signal was divided into epochs from 200 ms before to 700 ms after the target. Epochs were corrected relative to a 200 ms pre-stimulus baseline.

Effect onset was detected on the basis of 95% confidence intervals computed from 5000 bootstrap samples of single-average difference curves. Sampling points were considered as significant at the 5%-level, when upper and lower bound of the confidence band shared algebraic signs for an interval exceeding 10 ms. ERP amplitudes collapsed across sampling points in the epoch from 50 to 90 ms were examined in repeated measures analyses of variance (ANOVA). The Huynh-Feldt correction was applied to adjust degrees of freedom (rounded down) and *P*-values for violations of the sphericity assumption.

## Results

**Figure 2:**
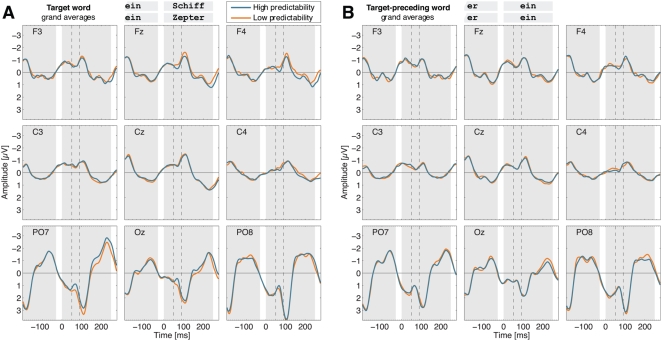
Grand averages for a sample of nine electrodes. ERPs for low (orange) and high predictability (blue) target conditions when (A) the target word or (B) the target-preceding word was presented. Background shading illustrates the stimulus sequence (gray: word present; white: blank screen). Dashed lines border the interval from 50 to 90 ms.

Grand average ERPs for low and high predictability target words are illustrated in Figure 2A for a sample of nine scalp electrodes. Curves are displayed for the interval from 200 ms before target onset up to the appearance of the target-succeeding word at 280 ms. Inspection of the data suggested amplitude differences at a surprisingly early latency – well before 100 ms. Amplitudes for high compared to low predictability words were more negative at posterior left locations and more positive at anterior right sites.

**Figure 3:**
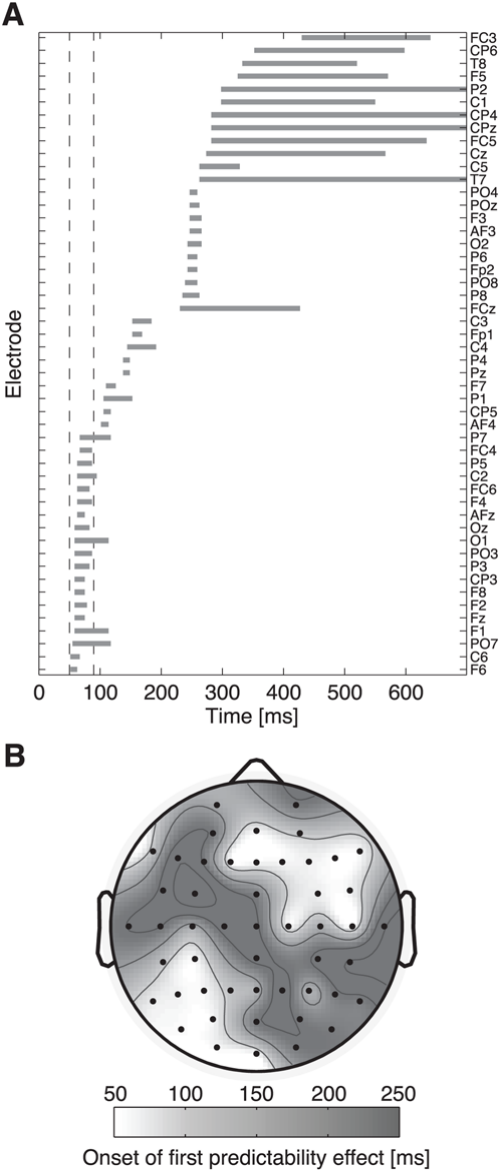
Latencies of the first predictability effect. (A) Gray bars illustrate onset and duration of the first significant predictability effect on 50 scalp electrodes. In the interval from 50 to 90 ms (dashed lines), the effect emerges on 19 channels. (B) The onset topography reveals early predictability effects at right anterior and left posterior sites.

The visual impression was corroborated in statistical analyses examining temporal onsets and durations of the first predictability effect (Figure 3A). From 0 to 100 ms, a total of 19 out of 50 scalp electrodes revealed significant amplitude differences with an average onset latency of 60 ms (SD: 4 ms) and a mean duration of 28 ms (SD: 16 ms). The earliest effect emerged at 52 ms post-stimulus. The topographical latency map (Figure 3B) identified early predictability effects at right anterior and left posterior sites.

Based on these results, we conducted statistical tests on mean amplitudes in the epoch from 50 to 90 ms after stimulus onset (dashed lines in Figure 3A). An ANOVA with frequency (2), predictability (2), and electrode (50) as within-subject factors yielded a main effect of electrode [*F*(2,70) = 14.66; *P*<.001; partial-η^2^ = .321] and, critically, an interaction of predictability×electrode [*F*(4,132) = 2.97; *P* = .019; partial-η^2^ = .098]. Neither the interaction of frequency×electrode (*P* = .298) nor the three-way interaction (*P* = .478) was significant (note that only interactions with the factor electrode are meaningful in this ANOVA because the average reference sets mean amplitudes across scalp channels to zero).

In order to strengthen evidence that the observed predictability effect was related to the experimental manipulation of targets, we examined ERPs for the two words prior to the target. These stimuli were identical across all conditions and were not subject to the predictability modulation from context sentences. Hence, amplitudes should not reveal any significant differences in the critical interval from 50 to 90 ms. ANOVAs with frequency (2), predictability (2), and electrode (50) as factors yielded no reliable effects for frequency, predictability, or the interaction of frequency×predictability (all *F*s<1). Additionally, ANOVAs on each of the two target-preceding words were performed in seven successive epochs of 40 ms, ranging from 0 to 280 ms after stimulus onset. None of these intervals revealed significant effects involving the factors frequency, predictability, or the interaction of frequency×predictability (all *P*s>.15). Grand average ERPs for the target-preceding word are displayed in Figure 2B.

**Figure 4:**
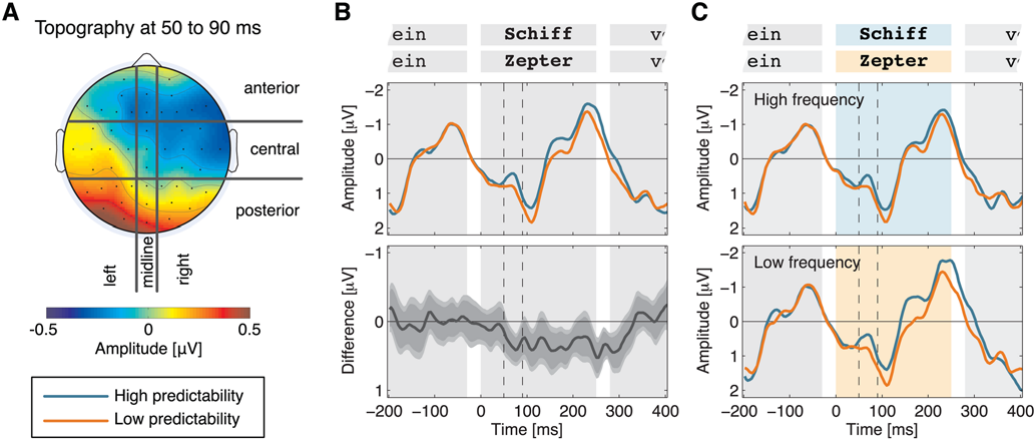
Predictability effect in scalp regions. (A) Topography of mean amplitude differences (low minus high predictability) in the epoch from 50 to 90 ms. Nine regions of scalp electrodes are delimited by black borders. (B) Mean amplitudes from seven electrodes at the posterior left region. In the interval from 50 to 90 ms (dashed lines), amplitudes are more negative for high (blue) than for low predictability (orange) words. The lower panel shows the difference waveform (low minus high predictability). Mid-gray and light-gray error bands depict 95% and 99% confidence intervals, respectively, computed from 5000 bootstrap samples. Background shading illustrates the stimulus sequence (gray: word present; white: blank screen). (C) Within-frequency class ERPs at the posterior left region. The early effect of predictability is independent from target frequency. Background shading reflects the stimulus sequence (shaded: word present; white: blank screen).

To scrutinize the predictability effect on the target word we grouped the 50 scalp electrodes into nine regions according to a grid of three sagittal (left, midline, right) and three coronal (anterior, central, posterior) fields (see Figure 4A). ERP amplitudes were collapsed across electrodes in corresponding regions and submitted to an ANOVA with the factors frequency (2), predictability (2), and region (9). The main effect of region [*F*(1,51) = 13.27; *P*<.001; partial-η^2^ = .300] and the interaction of predictability×region [*F*(2,80) = 3.36; *P* = .028; partial-η^2^ = .098] were significant. No other factors were statistically reliable (all *P*s>.15). Post-hoc two-way ANOVAs with the factors frequency (2) and predictability (2) in each of the nine regions yielded significant predictability effects at anterior-midline [*F*(1,31) = 4.47; *P* = .043; *partial-η^2^* = .126], anterior-right [*F*(1,31) = 4.73; *P* = .037; partial-η^2^ = .132], central-right [*F*(1,31) = 9.43; *P* = .004; *partial-η^2^* = .233], and posterior-left sites [*F*(1,31) = 10.67; *P* = .003; *partial-η^2^* = .256; shown in Figure 4B]. The main effect of frequency and the interaction of frequency×predictability were not reliable in any of the nine regions (all *P*s>.10).

Finally, we conducted separate analyses for low and high frequency words in the posterior-left region, which yielded the strongest effect (Figure 4A–C). As shown in Figure 4C, we consistently found more negative amplitudes for high than for low predictability words within the low frequency (*t*(31) = −2.25; *P* = .032) as well as within the high frequency condition (*t*(31) = −2.54; *P* = .016).

## Discussion

The present study examined the earliest index for the interplay between expectancy-based top-down and stimulus-driven bottom-up processes in sentence reading. ERPs to predictable and unpredictable words differed in an interval from 50 to 90 ms after stimulus onset, a latency that is considerably faster than most previous reports of interactions between top-down and bottom-up information in visual perception. It should be noted that other target properties cannot serve as an explanation for the effect because low and high predictability conditions utilized the same words in identical sentence frames; only preceding context sentences rendered targets expected or unexpected. Words prior to the target did not evoke differential ERPs across frequency and predictability conditions, corroborating the view that the observed effect resulted from the experimental manipulation of the target word. Importantly, the predictability effect held across levels of word frequency (Figure 4C) pointing to the reliability of the result. Furthermore, the independence from frequency rules out visual word familiarity as an explanation. We therefore propose that ERP differences have emerged from a rapid match of form-specific predictions with incoming visual patterns.

The finding contributes to the idea that active top-down predictions play a major role in early visual processing [2]–[4], [12], [23], [24], [26]–[30], [32]. As was noted previously, the large amount of feedback connections warrants projections to early cortical regions (e.g., [5]). Accordingly, fMRI studies have revealed top-down activations of primary sensory areas prior to the occurrence of expected stimuli [7], [9]. In visual word recognition, predictions were shown to pre-activate form-specific patterns of expected words (e.g., [35]). The present data indicate, that these predictions are verified very rapidly with the actual incoming stimulus, i.e., before 90 ms after visual onset.

Notably, the predictability effect occurred substantially earlier than in previous research. We consider two explanations why top-down effects at comparable latencies have been rarely reported before. First, we presume that powerful top-down projections are required to produce measurable influences at early latencies. In previous studies, effects potentially were indiscoverable or absent as a consequence of insufficiently strong feedback information. For example, effects of spatial attention were usually found from around 70 ms on P1 amplitudes, whereas the C1 component from 50 to 90 ms was unaffected [13]–[16]. However, variable SOAs inducing temporal uncertainty may have reduced the strength of attention towards upcoming stimuli. By contrast, with fixed SOAs and individual differences taken into account, attention effects on the C1 were found after 57 ms [40]. Beyond that, top-down influences vary in the amount of information they carry [4]. Feedback signals issuing spatial selection are presumably weaker than expectations pre-activating form-specific representations of predicted stimuli [35], [36]. The present data indicate that word predictability afforded top-down modulations that were strong enough to affect earliest perceptual processes.

As a second explanation, we presume that the observation of early top-down modulations depends on the perceptual task (see also [4]). In particular, early processes are enforced when task demands and perceptual load are sufficiently high [10], [33], [34]. In word recognition, normal reading speed of four to five words per second sets tight time constraints for stimulus processing. Compared to that, ERP reading experiments typically used slow rates of one or two words per second and potentially missed adequate demands. Those mostly revealed predictability effects from 200 to 500 ms on the N400 component [41]–[43]; only a few authors reported earlier effects, from 120 to 190 ms [44], [45]. Employing a quasi-normal reading speed, the present setup presumably approximated temporal conditions word recognition is optimized for and encouraged rapid integration of both top-down and bottom-up information. This is comparable to auditory sentence processing at normal speaking rate, where expected and unexpected inflections on adjectives evoked differential ERPs no later than after 50 ms [36].

These two proposals are neither exclusive nor exhaustive and, certainly, a number of additional factors will influence the timing of convergence between bottom-up and top-down streams in visual processing. To examine the validity of the present suggestions and to complete the picture of short-latency top-down effects, further research will be necessary. The reconciliation of these findings with feedback modulations occurring later in time will contribute to a comprehensive understanding about the interplay of internal brain states and information from the environment.

Clearly, the present data point to the efficiency of stimulus encoding in visual perception. Evidence from electro- and magneto-encephalography revealed that bottom-up activation spreads in the primary visual cortex at around 50 ms post-stimulus and is rapidly transmitted to higher cortical areas. Activity reaches a large proportion of extrastriate and frontal regions within 70 and 80 ms, respectively [46], [47]. Can these signals be interpreted and compared to stored information before 90 ms? Converging empirical support comes from visual search. Sigman and colleagues showed that extensive training with letter-like shapes grants selective responsiveness in earliest visual cortices [32]. Further, complex search patterns that were either predictive or unpredictive with respect to target position evoked differential magneto-encephalographic responses from 50 to 100 ms at occipital sites. Since participants were not aware of the pattern-target associations, this result points to fast elaboration of visual input that rapidly contacts unconscious memory [48]. An explanation for the high processing speed of visual input is provided by recent theories proposing that meaningful information is already extracted from the first 1–5% of the bottom-up signal. Thereby, top-down processes, acting as temporal bias, increase stimulus saliency [49], [50]. Consistent with these ideas, our data indicate that in the presence of strong predictions, the cortex matches pre-activated representations with incoming stimuli shortly after the visual signal is available.

This interpretation is in line with models assuming interactions between feedforward and feedback information (e.g., [23], [24], [26]–[30]). For instance, Di Lollo and co-workers [27] proposed that early visual processes generate preconscious hypotheses about the identity of an incoming stimulus. These hypotheses re-enter low visual areas and are iteratively compared with the input. An affirmative match enhances the signal and affords conscious perception of a stimulus. This interactive view of feedforward and feedback information successfully accounted for findings from backward masking, assuming that top-down hypotheses from a briefly presented target mismatch the visual input after a mask has superseded the bottom-up target signature [27], [51]. Further, rapid resumption of an interrupted visual search indicates that preprocessed patterns evoke target-specific hypotheses which are swiftly tested against sensory information [52], [53]. The present data extend this view suggesting that top-down hypotheses also emerge from the interpretation of semantic contexts. Thereby, the instantaneous match with the visual input is compatible with the idea that top-down influences dynamically reconfigure filters in the visual system to grant optimal processing of relevant information from incoming signals [54]. Thus, visual perception appears as an active process that rapidly compares internal semantic representations with task-relevant aspects of incoming stimuli [55]–[57].

The observed predictability effect was strongest over posterior electrodes. This region is situated above the left hemispheric occipito-temporal network that is strongly linked to the so-called visual word form area [58], [59]. As these cortical structures are gradually sensitive to the processing of word-like stimuli [31], they reflect a plausible ground for the matching of top-down predictions and incoming signals. Another relevant structure may be the foveal portion of the retinotopic cortex that was shown to receive category-specific feedback information as response to peripherally presented objects. Hence, V1 was proposed to serve as scratch pad for the storage and computation of task-relevant visual information [60] (see also [4]). Note, however, that suggestions about underlying sources of the predictability effect remain speculative, as no strong inferences about localization can be drawn on the basis of the present ERP data.

In conclusion, previous research has shown that predictions about upcoming words pre-activate representations of specific word forms. The present results indicate that, under near-normal reading speed, these predictions are checked in an interval from 50 to 90 ms after the visual input. Though reading is ideally suited to examine this issue, rapid verification of expected physical input is fundamental to many domains, including object recognition in general [5] and movement control [61]. If replicable across a wide range of tasks, our finding provides a critical temporal constraint for theories of top-down and bottom-up interactions as well as novel insights about the efficiency of stimulus encoding.
